# Design and Processing of Gas Turbine Blades Based on Additive Manufacturing Technology

**DOI:** 10.3390/mi14091675

**Published:** 2023-08-27

**Authors:** Xuan Liu, Xingguo Han, Guofu Yin, Xiaohui Song, Lixiu Cui

**Affiliations:** 1 School of Mechanical and Electrical Engineering, Guilin University of Electronic Technology, Guilin 541004, China; 17863642183@163.com; 2 School of Mechanical Engineering, Guilin University of Aerospace Technology, Guilin 541004, China; sxhui@guat.edu.cn (X.S.); cuilixiu@guat.edu.cn (L.C.); 3 School of Mechanical Engineering, Sichuan University, Chengdu 610065, China; gfyin@scu.edu.cn

**Keywords:** additive manufacturing, gas turbine blade, surface fitting, investment casting, fused deposition

## Abstract

Aiming at the problems of the complex shape, difficult three-dimensional (3D) digital modeling and high manufacturing quality requirements of gas turbine blades (GTB), a method of fitting the blade profile line based on a cubic uniform B-spline interpolation function was proposed. Firstly, surface modeling technology was used to complete the fitting of the blade profile of the GTB, and the 3D model of the GTB was synthesized. Secondly, the processing parameters of the additive manufacturing were set, and the GTB model was printed by fused deposition technology. Then, the rapid investment casting was completed with the printed model as a wax model to obtain the GTB casting. Finally, the blade casting was post-processed and measured, and it was found to meet the requirements of machining accuracy and surface quality.

## 1. Introduction

In recent years, additive manufacturing (3D printing) has played an important role in the processing and production of parts. There has been a significant amount of research, such as path planning [1], research on unsupported printing [2], improvements in stiffness and the stability of parts [3], multi-mode printing [4] and other aspects [5].

GTB manufacturing has been the key technology for the development of power equipment in the past 50 years. It is the integration of mechanical, material, manufacturing, surface science and technological achievements. The development of blades has improved the efficiency of aircraft engines [6] and gas turbines. Blades have high technical added value [7]. Because of the complex shape of the blade, GTB manufacturing quality requirements are extremely high.

GTB have complex surfaces [8,9,10,11], and their 3D digital modeling process is complex. The modeling effect has a great influence on the manufacture, analysis and application of the blade [12,13,14]. In order to obtain a better blade modeling effect and simplify the modeling process, significant research has been conducted on blade modeling.

According to the special requirements of compressor rotor blade design, CAD software engineering regulations, and specific software and hardware environments, Li et al. [15] adopted the system development method combining the life cycle method and rapid prototyping method and established a blade CAD/CAM special system on the UG platform. For the blade precision forging process, Zhao [16] developed a special CAD system for the precision forging of blades. The system can automatically achieve the original data transformation of the blade surface and the solid modeling of the blade body. It can recall the birch head and boss entity from the feature library and automatically generate functional modules, such as component mold surfaces and blade hair edge grooves. Xia [17] focuses on aircraft engine turbine blades. He studied the key technologies of the turbine blade modeling CAD system, including the turbine blade shape modeling method comprising the turbine blade feature information model, the organic unity of the turbine blade design information and geometric information. Wang et al. [18] studied the 3D modeling method for turbine blades. Based on the free-form surface modeling function of UGNX, they used the bicubic B-spline fitting method to establish the spatial spline curve model of the blade surface and constructed the spatial surface of the blade. At the same time, they analyzed several key points on the blade surface modeling mathematically and verified the feasibility of the modeling method. In view of the complexity and irregularity of the blade structure of an aircraft engine, Yang et al. [19] used UG to study how to make the spline curve corresponding to the blade profile become a smooth surface and damping table after lofting. Taking a certain type of aircraft engine blade as an example, they analyzed several key problems in blade modeling and verified the feasibility of the method. According to the structural characteristics of steam turbine blades, Yu et al. [20] divided the blade into a root, root-surface, crown, crown-surface and body. Among these, the blade body can be divided into the back arc surface, inlet arc surface, inner arc surface and outlet arc surface. According to these characteristics, they used UG to develop a blade modeling module that can model each feature according to the blade parameters input by the user. This module can generate a 3D model of the blade for the development of blade processing software. Based on the feature modeling method, Zhang et al. [21] developed the design of the parametric parts of turbine blades. They proposed a method for establishing a library of turbine blade parts for turbine design and optimization. This method reduces the workload in the process of turbine blade design and optimization. They presented a specific method for parametric turbine blade parts that can effectively improve the efficiency of turbine blade modeling.

According to the parameters of GTB, the blade profile was fitted by a cubic uniform B-spline interpolation function. The surface modeling technology of UG was used to complete the fitting of the blade profile and generate the 3D model of the GTB. The model of the GTB was printed by fused deposition additive manufacturing technology [22], and the model was used as the wax model of the GTB to complete rapid investment casting to obtain the blade casting.

## 2. The Parameters of GTB

**Figure 1:**
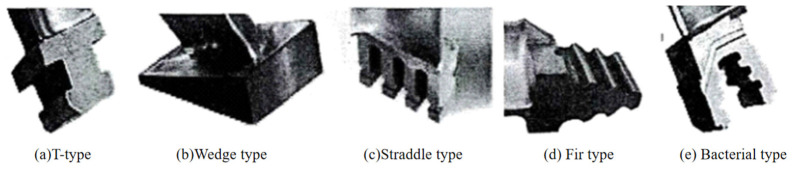
Tenon structures.

The GTB is mainly composed of the blade root, blade body and blade crown. The blade root and the rotor hub are installed on the rotor. The blade profile is the channel of airflow, and its shape characteristics have a great influence on the efficiency of a steam turbine. The blade crown is the part of the blade. It is also known as the tenon. It is the main part of the GTB. According to the shape of the cross section, the commonly used blade tenons can be divided into T-type, Wedge type, Straddle type, Fir type, Bacterial type, etc., as shown in Figure 1.

**Figure 2:**
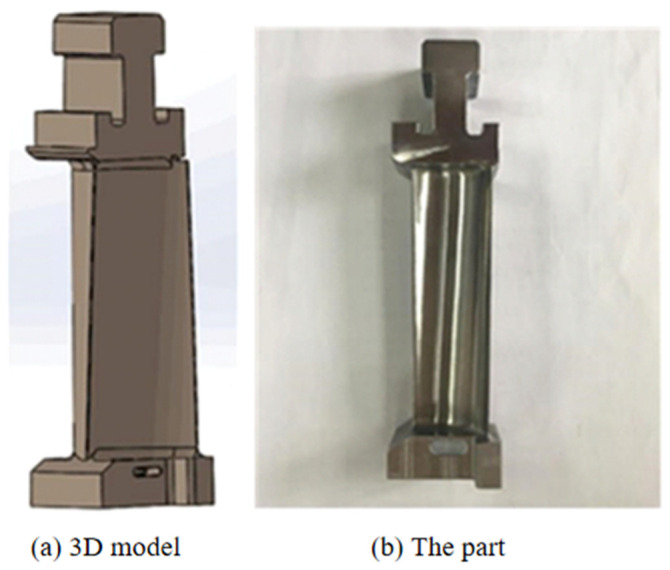
Gas turbine blade.

**Figure 3:**
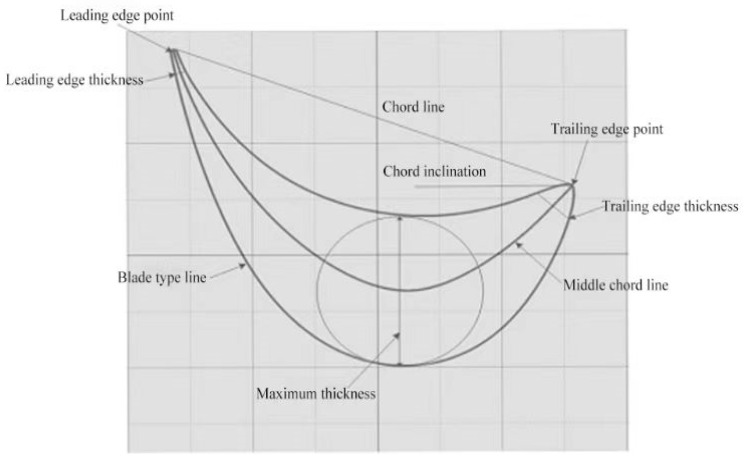
Characteristic parameters of the blade’s molded surface.

A gas turbine blade produced by an enterprise is shown in Figure 2. The characteristic parameters of the blade profile are the most complex. As shown in Figure 3, the blade profile is composed of complex curved lines. The curvature of the leading and trailing edge points of the blade profile is large, which is in the flow channel of the gas turbine. According to the analysis and calculation of aerodynamic data, the cross sections along different blade heights are obtained. According to the specific cross-sectional area superposition law and distortion law, these cross sections form a spatial surface. The blade body is formed by the blade type in the direction of the blade height, according to the specific stacking law and twisting law. The blade profile is the profile of the blade in the blade height direction, which is calculated based on aerodynamic data. During the design of GTB, after obtaining the coordinate point data of the aerodynamic design, the cross-section point data is fitted according to a certain algorithm to form a smooth blade cross-section line. Secondly, the fitted section line group is fitted according to a certain method to form the blade surface. The molding process generates the blade surface in a point–line–surface manner. Finally, a blade body model is formed.

In this paper, the blade’s body formation is studied based on the point cloud data of the blade’s body surface. The blade’s surface formation method is also suitable for obtaining the point cloud data of the blade’s surface by reverse technology. These data can be used to form the blade’s surface.

## 3. Blade Profile Modeling of GTB

The formation of the GTB body is mainly completed in two steps. The first step is to use point data fitting to form the blade section line. Because there are several point data of cross-section lines in the blade design, these point data are fitted by the same method to generate the cross-section line group. The second step is to generate the blade profile according to a certain fitting method, and then smooth the profile to obtain the required blade model.

The cross-section point cloud data of the GTB is stored in a *.TXT file. At present, the commonly used curve fitting methods are parametric cubic spline curve fitting, Bezier curve fitting, non-uniform rational B-spline curve fitting, etc. In this paper, the cubic uniform B-spline interpolation function is used to fit the blade profile.

Given n + 1 control points $V_{i+j}\left(j=0,1,2,\ldots ,n\right)$ of the *k*-th joint, the *r*-th B-spline function can be defined as follows:

(1) $$P_{i}\left(u\right)=\sum_{i=0}^{n}B_{i,r}\left(u\right)V_{i+j}$$

$B_{i,r}\left(u\right)$ is the basis function of the B-spline function of degree *r*, which can be obtained by the following recursive formula (De Boor recursive formula [23]).

(2) $$\{\begin{array}{l} B_{i,0}\left(u\right)=\{\begin{array}{l} 1,\;u\in \left[u_{i},u_{i+1}\right]\\ 0,\;otherwise \end{array}\\ B_{i,r}\left(u\right)=\frac{u-u_{i}}{u_{i+r}-u_{i}}B_{i,r-1}\left(u\right)+\frac{u_{i+k+1}-u}{u_{i+r+1}-u_{i+1}}B_{i+1,r-1}\left(u\right),r\geq 1\\ \frac{0}{0}=0 \end{array}$$

The order of the B-spline has a great influence on the performance of the trajectory. The low-order smoothness is not good, and the high-order can easily cause oscillation. Therefore, the cubic B-spline function is selected. When the order is 3, the *i*-th trajectory can be expressed as follows:

(3) $$B_{i,r}\left(u\right)=\frac{u-u_{i}}{u_{i+3}-u_{i}}B_{i,\;2}\left(u\right)+\frac{u_{i+4}-u}{u_{i+4}-u_{i+1}}B_{i+1,\;2}\left(u\right)$$

Expanded by intervals, the Formula (3) becomes as follows:

(4) $$B_{i,3}\left(u\right)=\{\begin{array}{c} \frac{u-u_{i}}{u_{i+3}-u_{i}}\cdot \frac{u-u_{i}}{u_{i+2}-u_{i}}\cdot \frac{u-u_{i}}{u_{i+1}-u_{i}}\;u\in \left[u_{i},u_{i+1}\right]\\ \frac{u-u_{i}}{u_{i+3}-u_{i}}\cdot \frac{u-u_{i}}{u_{i+2}-u_{i}}\cdot \frac{u_{i+2}-u}{u_{i+2}-u_{i+1}}+\frac{u-u_{i}}{u_{i+3}-u_{i}}\cdot \frac{u_{i+3}-u}{u_{i+3}-u_{i+1}}\cdot \frac{u-u_{i+1}}{u_{i+2}-u_{i+1}}+\frac{u_{i+4}-u}{u_{i+4}-u_{i+1}}\cdot \frac{u-u_{i+1}}{u_{i+3}-u_{i+1}}\cdot \frac{u-u_{i+1}}{u_{i+2}-u_{i+1}}\;u\in \left[u_{i+1},u_{i+2}\right]\\ \frac{u-u_{i}}{u_{i+3}-u_{i}}\cdot \frac{u_{i+3}-u}{u_{i+3}-u_{i+1}}\cdot \frac{u_{i+3}-u}{u_{i+3}-u_{i+2}}+\frac{u_{i+4}-u}{u_{i+4}-u_{i+1}}\cdot \frac{u-u_{i+1}}{u_{i+3}-u_{i+1}}\cdot \frac{u_{i+3}-u}{u_{i+3}-u_{i+2}}+\frac{u_{i+4}-u}{u_{i+4}-u_{i+1}}\cdot \frac{u_{i+4}-u}{u_{i+4}-u_{i+2}}\cdot \frac{u-u_{i+2}}{u_{i+3}-u_{i+2}}\;u\in \left[u_{i+2},u_{i+3}\right]\\ \frac{u_{i+4}-u}{u_{i+4}-u_{i+1}}\cdot \frac{u_{i+4}-u}{u_{i+4}-u_{i+2}}\cdot \frac{u_{i+4}-u}{u_{i+4}-u_{i+3}}\;u\in \left[u_{i+3},u_{i+4}\right] \end{array}$$

The above is the general expression of B-spline. In this paper, the cubic uniform B-spline is used, and the interval is [0,1]. Its expression is as follows:

(5) $$\{\begin{array}{l} B_{0,\;3}=\frac{1}{6}{\left(1-u\right)}^{3}\\ B_{1,\;3}=\frac{1}{6}\left(3u^{3}-6u^{2}+4\right)\\ B_{2,\;3}=\frac{1}{6}\left[-3u^{3}+3u^{2}+3u+1\right]\\ B_{3,\;3}=\frac{1}{6}u^{3} \end{array}\;u\in \left[0,1\right]$$

The matrix form of Formula (1) is:

(6) $$P_{i}\left(u\right)=\sum_{i=0}^{3}B_{i,\;3}\left(u\right)V_{i+j}=\frac{1}{6}\left[\begin{array}{cccc} u^{3} & u^{2} & u & 1 \end{array}\right]\left[\begin{array}{cccc} -1 & 3 & -3 & 1\\ 3 & -6 & 3 & 0\\ -3 & 0 & 3 & 0\\ 1 & 4 & 1 & 0 \end{array}\right]\left[\begin{array}{c} V_{i}\\ V_{i+1}\\ V_{i+2}\\ V_{i+3} \end{array}\right],u=\left[0,1\right],j=0,1,2,3$$

From Formula (6), the properties of its endpoints are discussed, and the geometric properties of any cubic B-spline can be obtained. The geometric properties can be expressed by the two methods in the following diagrams.

**Figure 4:**
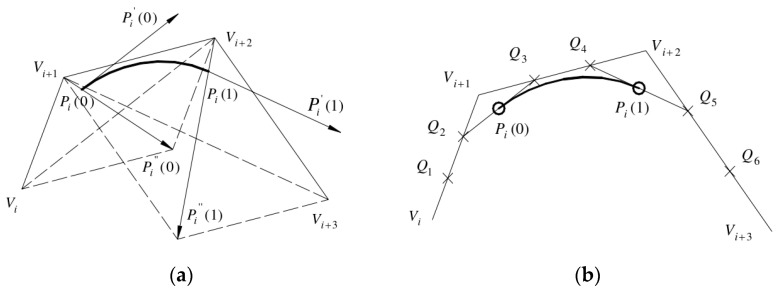
Geometric properties of cubic uniform B-spline curves. (**a**) Common geometric representation; (**b**) Equivalent representation.

Figure 4a is a common geometric representation of cubic uniform B-spline curve segments. As can be seen from the diagram; let u be equal to 0 and 1, respectively, and the coordinates of $P_{i}\left(0\right)$ and $P_{i}\left(1\right)$ points can be obtained. The formula is as follows:

(7) $$\{\begin{array}{l} P_{i}\left(0\right)=\frac{1}{6}\left(V_{i}+4V_{i+1}+V_{i+2}\right)\\ P_{i}\left(1\right)=\frac{1}{6}\left(V_{i+1}+4V_{i+2}+V_{i+3}\right)\\ \dot{P_{i}}\left(0\right)=\frac{1}{2}\left(V_{i+2}-V_{i}\right)\\ \dot{P_{i}}\left(1\right)=\frac{1}{2}\left(V_{i+3}-V_{i+1}\right)\\ \overset{..}{P}\left(0\right)=V_{i}-2V_{i+1}+V_{i+2}\\ \overset{..}{P}\left(1\right)=V_{i+1}-2V_{i+2}+V_{i+3} \end{array}$$

Point $P_{i}\left(0\right)$ is located on the midline of triangle $V_{i}V_{i+1}V_{i+2}$, and line $V_{i+1}P_{i}\left(0\right)$ is one-third of its midline. Point $P_{i}\left(1\right)$ is located on the midline of triangle $V_{i+1}V_{i+2}V_{i+3}$, and line $V_{i+2}P_{i}\left(1\right)$ is one-third of its midline. It can also be seen from the figure that the uniform B-spline curve does not pass through the control points. Point $P_{i}\left(0\right)$ is only related to the first three control points, and point $P_{i}\left(1\right)$ is only related to the last three control points. In fact, the uniform B-spline is closely influenced by this control point, which is the reason for the good local adjustment of the uniform B-spline. The cubic uniform B-spline can achieve second-order continuity.

In Figure 4b, the three edges of the feature polygon composed of the four feature points defining the line segment are divided into three equal parts, points $Q_{1}$ to $Q_{6}$ are the corresponding equal points. The middle point of segment $Q_{2}Q_{3}$ and segment $Q_{4}Q_{5}$ are the starting point of the curve segment $P_{i}\left(0\right)$, the end point is $P_{i}\left(1\right)$, respectively. And the tangent vectors of the first and end points of the curve segment are $Q_{2}Q_{3}$ and $Q_{4}Q_{5}$, respectively. A cubic uniform B-spline curve segment defined by control points $V_{i}$, $V_{i+1}$, $V_{i+3}$ and $V_{i+3}$ is equivalent to a cubic Bezier curve defined by points $P_{i}\left(0\right)$, $Q_{3}$, $Q_{4}$ and $P_{i}\left(1\right)$. It can be proved by the following formula that the geometric meaning of Figure 4a,b is the same.

(8) $$\mathrm{By}\{\begin{array}{l} Q_{2}=\frac{1}{3}V_{i}+\frac{2}{3}V_{i+1}\\ Q_{3}=\frac{2}{3}V_{i+1}+\frac{1}{3}V_{i+2}\\ Q_{4}=\frac{1}{3}V_{i+1}+\frac{2}{3}V_{i+2}\\ Q_{5}=\frac{2}{3}V_{i+2}+\frac{1}{3}V_{i+3} \end{array},\;\mathrm{then}$$

(9) $$\{\begin{array}{l} P_{i}\left(0\right)=\frac{1}{2}\left(Q_{2}+Q_{3}\right)=\frac{1}{6}\left(V_{i}+4V_{i+1}+V_{i+2}\right)\\ P_{i}\left(1\right)=\frac{1}{2}\left(Q_{4}+Q_{5}\right)=\frac{1}{6}\left(V_{i+1}+4V_{i+2}+V_{i+3}\right)\\ \dot{P_{i}}\left(0\right)=3\left(Q_{3}-P_{i}\left(0\right)\right)=\frac{1}{2}\left(V_{i+2}-V_{i}\right)\\ \dot{P_{i}}\left(1\right)=3\left(P_{i}\left(1\right)-Q_{4}\right)=\frac{1}{2}\left(V_{i+3}-V_{i+1}\right) \end{array}$$

**Figure 5:**
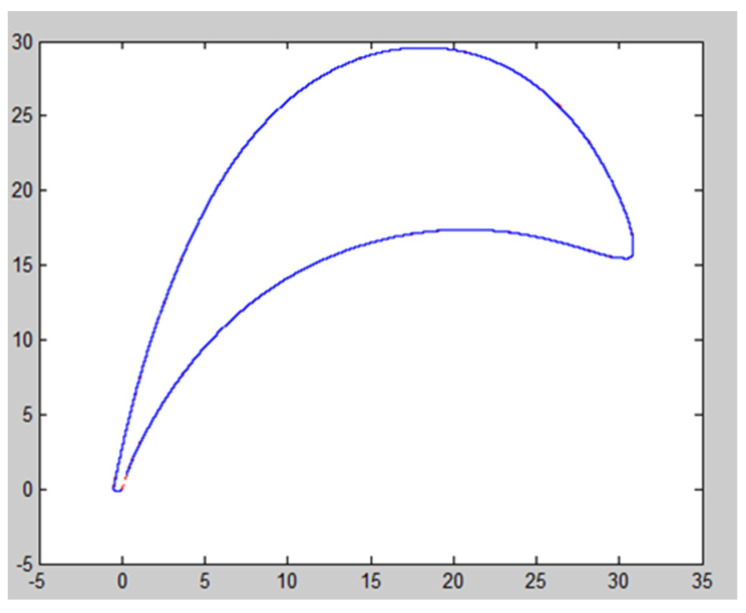
B-spline curve fitting.

According to the B-spline curve function, the program is written in MATLAB. The original point from the *.TXT file is read, and the original data is used to inverse the control points. The control points are used to fit the cubic uniform B-spline curve so that the curve passes through the original point until the two vertices of the first and last positions of the fitted curve coincide [24]. Thus, the spline curve based on the point cloud data can be drawn. According to its functional characteristics, the shape control boundary conditions are studied, and the conclusion is drawn: the closed curve is formed by point data fitting, and the four pairs of point data at the first and last positions are required to coincide (the coincidence of two vertices can ensure that the B-spline curve segment is tangent to the regular polygon; the four pairs of point data at the first and last positions are required because the control polygon of each curve segment of the cubic B-spline is composed of four vertices). The B-spline curve generated by the point data is shown in Figure 5.

Through MATLAB, the fitting curve is obtained by using the cubic uniform B-spline, and the measurement error is obtained by the following method: ten X coordinates are selected, the Y value corresponding to each X value on the fitting curve is compared with the Y value corresponding to the coordinate on the real curve, and the deviation value can be obtained. The deviation value can reflect the fitting error.

It is found that the deviation values are different at different positions of the leaves. The fitting deviations at the leading-edge point and the trailing edge point are large, and the deviation range is from 0.002 mm to 0.020 mm. The greater the curvature of the curve, the greater the fitting deviation value. The deviation of the blade profile fitting is small, ranging from 0.002 mm to 0.010 mm. The size of the fitting deviation value is related to the number of point cloud data. The higher the number of point cloud data, the smaller the deviation value and the better the fitting effect. The lower the number of point cloud data, the greater the deviation value and the worse the fitting effect.

UG/Open Grip is used to create the blade profile line, and the blade profile is generated according to the generated profile line. The B-curve is generated by a series of points, or these points are used as control points to generate the B-curve, and the point method is used to generate the B-curve to create the blade section line.

According to the known points, the blade section line group is obtained, and the fitting of the blade surface is completed by UG. There are four main types of B-surface modeling in UG: the point method to generate the B-surface, the curve method to generate the B-surface, the quadric surface method to generate the B-surface and the curve grid method to generate the B-surface. According to the characteristics of the curve group and the forming characteristics of the blade surface in this paper, the curve method is selected to generate the blade surface; that is, the blade surface is generated by a set of blade section curves.

**Figure 6:**
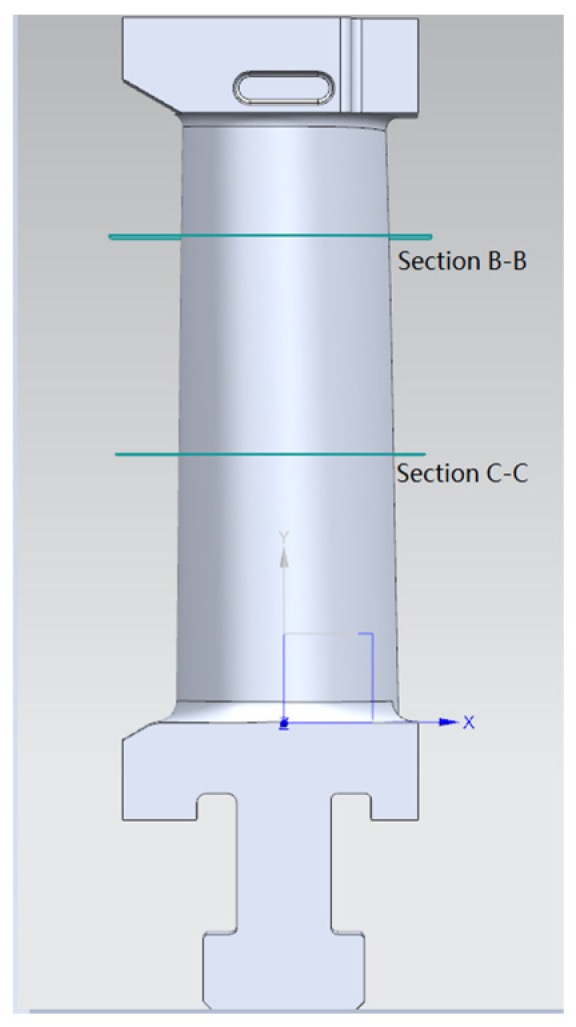
Complete blade model.

In order to facilitate the Boolean summation of the blade crown, tenon and blade body, the same coordinate system is used in the design of the parametric module of the blade crown, tenon and blade body. In this way, in the same coordinate system, the leaf crown, tenon and body can be obtained respectively, and the complete 3D model of the leaf can be obtained by Boolean summation, as shown in Figure 6.

## 4. Additive Manufacturing of GTB

Solid models of various materials can be printed by fused deposition additive manufacturing equipment. The most widely used material is ABS. ABS is a thermoplastic polymer material with high strength, good toughness and easy processing, also known as ABS resin. In this paper, the Uprint SE equipment produced by Stratasys is used to print the blade model of ABS material.

**Figure 7:**
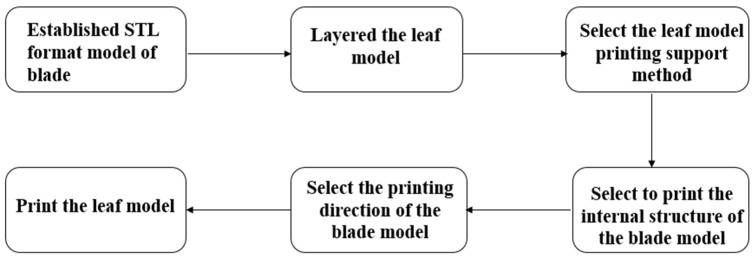
Blade model printing process.

**Figure 8:**
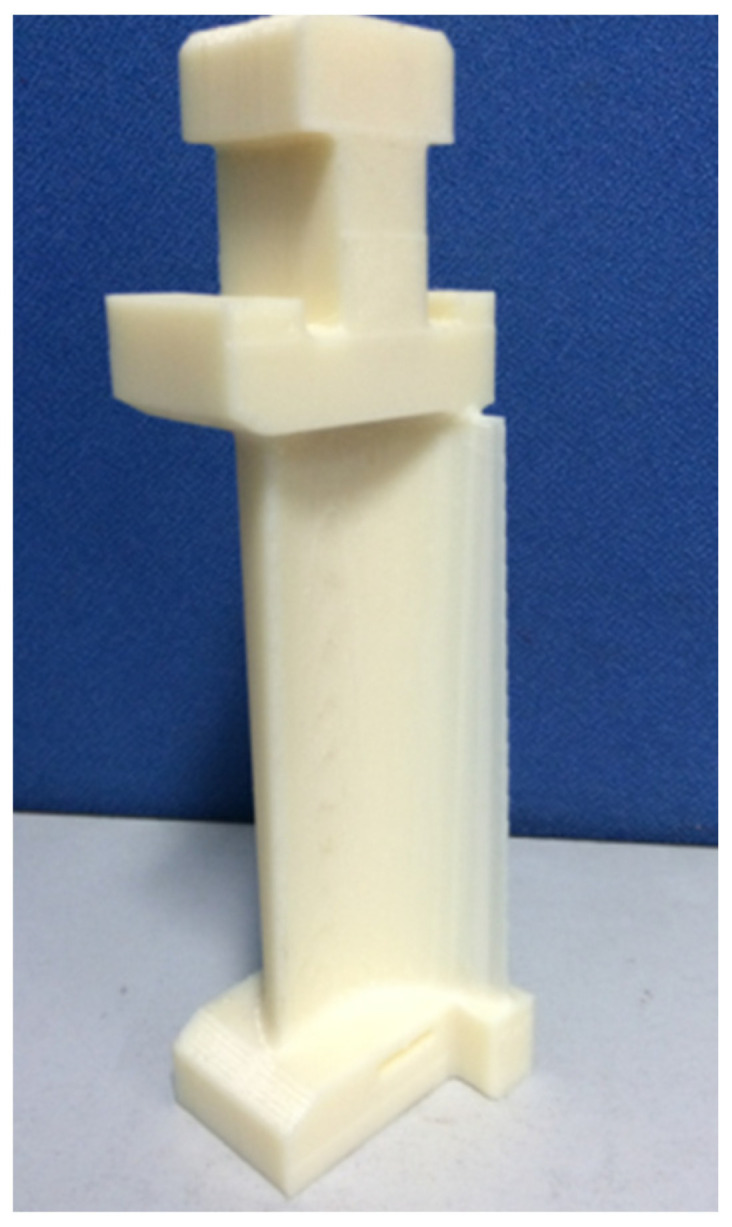
Gas turbine additive manufacturing model.

The blade model is processed by additive manufacturing technology. Firstly, the blade model needs to be established in the 3D modeling software and converted into an STL format. An STL file is the standard data format for describing additive manufacturing models. The ability to slice and layer the 3D model is the premise of the additive manufacturing function. The model is imported into the slicing software for slicing. The key to the slicing processing is to select the printing direction and support mode of the blade model and the internal model structure of the printing blade. Then, the blade model is layered after setting the additive manufacturing parameters. The slicing algorithm based on the STL model is adopted. All the planes intersect with the part model, and the intersection points of the tangent plane and all the triangular facets are obtained. The contour formed by connecting each intersection point, in turn, with a straight line is the section contour of the layer. For general regular parts, the cross-section contour has little error compared with the actual contour of the layer, but, for some complex surface parts, the surface part should have higher conversion accuracy than the plane part. The traditional slicing method based on the STL model makes it easy to cause contour distortions at different levels for complex curved surface parts. In this paper, a layered section generation algorithm for complex curved surface parts based on the NURBS curve is adopted when layered based on the STL model [25]. Finally, the additive manufacturing equipment is used to print the blade model. The whole process is shown in Figure 7. The additive manufacturing parameters are set, as shown in Table 1. The GTB printing model is shown in Figure 8.

## 5. Investment Casting of GTB Based on FDM

### 5.1. Investment Casting of Blade

The traditional blade investment casting generally uses the designed blade master pattern to press the blade wax model, and then the blade wax model is bonded to the casting system, according to the set casting scheme, to form a wax model module. The wax model module is made by sanding and other processes. After the shell is dewaxed in the dewaxing kettle, the shell is roasted in the baking furnace, and the required blade casting parts can be obtained by pouring the liquid metal. In this paper, the blade model of ABS resin material made by FDM additive manufacturing technology is proposed to replace the blade wax model in the traditional casting process to complete the investment casting of the blade.

In order to reduce the influence of thermal expansion of the ABS material blade pattern on the shell and improve the dimensional accuracy of blade products, the shell will be broken if the effect is serious. Firstly, the dewaxing treatment is carried out in a high-pressure dewaxing kettle. The high-pressure dewaxing kettle has a pressure protection function. The protection pressure is 0.8 MPa, the dewaxing time is set to 20 minutes, and the dewaxing temperature is controlled at about 168 °C. After dewaxing, the ABS material is heated in a baking furnace. Because there is no pressure protection when heating in the baking furnace, in order to prevent the shell from bursting during the baking process, the temperature is slowly and continuously loaded to ensure that the ABS material is continuously removed from the ingate after melting, and the complete shell is obtained after cleaning. After firing, the shell is put into the sand, and the liquid metal is poured. The casting grade is A297HK-type heat-resistant cast steel material. During the pouring process, the shell does not crack. After pouring, the casting is cooled for a certain time and then cleaned to obtain a complete casting part. The test finds that the external dimensions of the casting are complete. In many experiments, there are a few small defects on the surface of castings. A possible reason is that there are some defects in the surface quality of the 3D-printed model, with more residual impurities of ABS material during the firing process.

**Figure 9:**
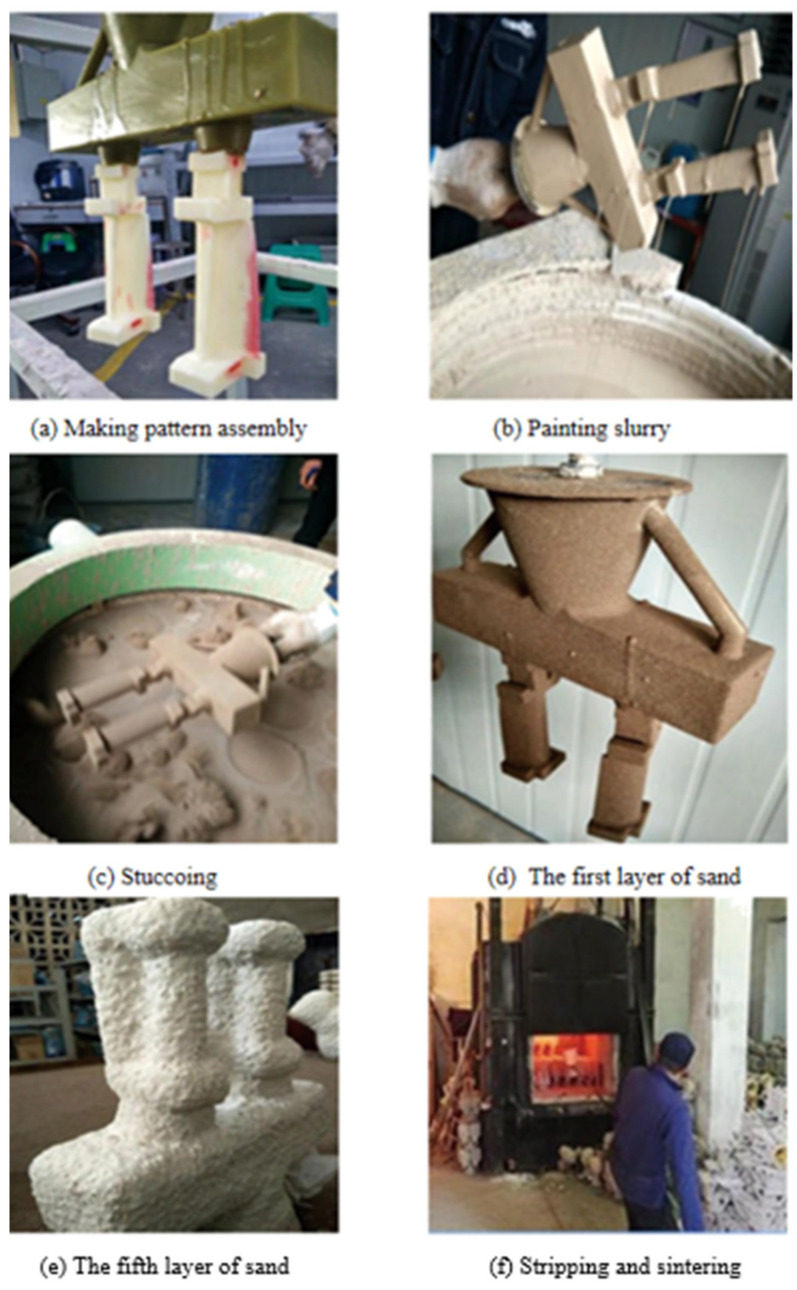
Rapid investment casting process route of the blade.

**Figure 10:**
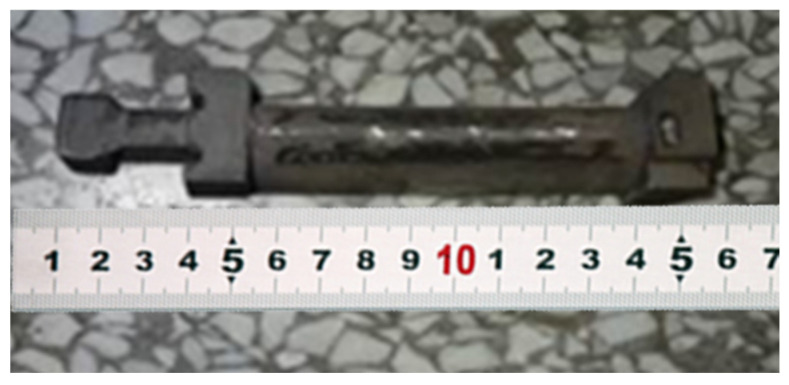
Casting part of the blade.

In the process of investment casting, a problem in the pouring system may lead to the thermal expansion of the blade model and the expansion of the shell in the process of pouring the liquid metal. In this experiment, the wax pouring system is optimized as follows. The optimized wax gating system is shown in Figure 9. Firstly, the GTB casting pattern is printed and processed. Secondly, the blade pattern and the casting system form a casting module. Thirdly, the module is coated and hung to dry multiple times with layers of sand, forming the shell. Fourthly, the module is dewaxed and defoliated after the sanding, hanging and roasting of the shell. Finally, the blade castings are formed by the casting, as shown in Figure 10.

(1) The 1 # and 2 # blade patterns are bonded on the same wax pouring system, and the design pouring method is stepped; that is, the metal liquid is injected from the lower part of the mold cavity during the pouring and gradually fills the mold cavity. The step-by-step method is beneficial to the smooth flow of the molten metal and effectively avoids the possibility of splashing, eddy currents, gas involvement, impurities and defects caused by top-injected molten metal.

(2) The ingate and riser are designed at the hot spot of the casting so that the casting feeding system stores enough liquid metal to supply the casting feeding, which can effectively avoid the loose defects of the internal structure after the forming of the parts.

(3) Using the ribbed gate cup to strengthen the connection between the gate cup and the sprue can prevent the formation of eddy currents in the molten metal. At the same time, it is not easy to drop the sand falls than the straight-edge gate cup, which can prevent casting defects caused by the falling of sand from the shell into the cavity in the later stage.

After obtaining the casting of the GTB, abrasive flow polishing technology is used to post-process the blade. Firstly, the roughness detector is used to detect the surface roughness of the blade to determine the time, pressure and abrasive particle size for the abrasive flow polishing. After polishing, the blade is taken out, and the surface residue is cleaned with an air gun with a pressure of 0.6 MPa. Then, the roughness detector is used to ensure that the surface finish of the blade meets the requirements. The biggest difference between traditional mechanical or manual polishing and abrasive flow polishing is the controllability and uniformity of abrasive flow polishing for accuracy, whereas the traditional mechanical or manual methods use an abrasive belt medium to grind repeatedly, and the polished texture is disorderly. Its uniformity and precision controllability cannot be guaranteed.

### 5.2. Experiments of Blade

**Figure 11:**
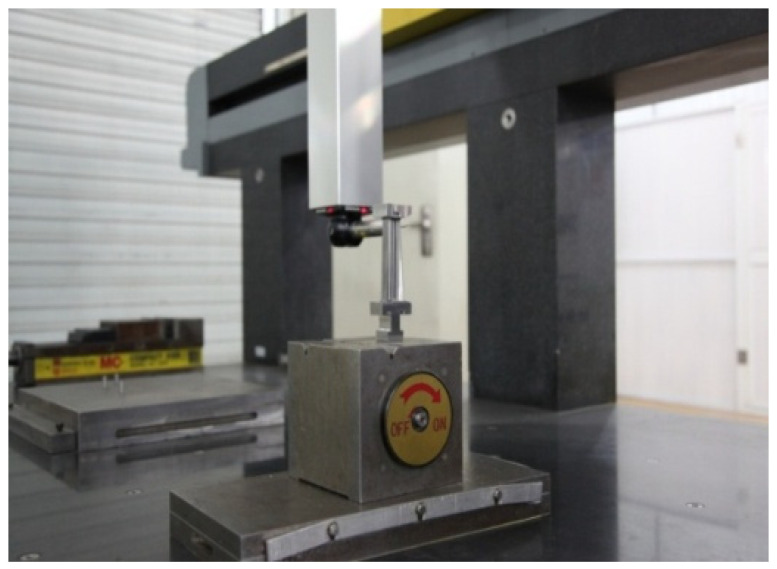
Blade profile measurement.

After the post-processing of the blade casting, the three-coordinate measuring machine is used to detect the blade surface [26], as shown in Figure 11. The measuring instrument is the ZOOL3 bridge measuring machine produced by Hexcon. During the blade measurement experiment, two specific points of cross-section data, section B-B with a distance of +32.5 mm from the horizontal plane of the blade root end face and section C-C with a distance of +65.00 mm, are collected, as shown in Figure 6. A profile deviation analysis of the two specific sections is carried out, and the profile tolerance range is from −0.1 mm to 0.1 mm, as shown in Table 2.

The profile deviation range of section B-B is from −0.081 mm to 0.086 mm, and the standard deviation is 0.043 mm; the profile deviation value range of section C-C is from −0.088 mm to 0.090 mm, the standard deviation value is 0.057 mm, and the known blade profile tolerance range is from −0.1 mm to 0.1 mm. It can be seen that the profile deviation values of the two key sections meet the requirements, which indicates that the rapid investment casting and GTB casting based on fused deposition additive manufacturing technology meet the requirements in terms of contour accuracy.

## 6. Conclusions

Aiming at the problems faced by the manufacturing of GTB, this paper proposes a design and processing method for GTB based on fused deposition additive manufacturing technology. This method improves the production efficiency, dimensional accuracy and surface quality of GTB. The main contents are as follows:

1. UG was used to extract the blade profile data from the original 3D model of the blade. Through MATLAB, the cubic uniform B-spline interpolation function was used to optimize the blade surface line of the gas turbine blade, and the data of the optimized blade surface line was obtained. UG was used to complete the modeling of the 3D model of the blade. These improved the accuracy of the 3D model of the blade.
2. The blade model was printed by fused deposition additive manufacturing technology. The model was used to replace the wax mold for investment casting. The investment casting process of the blade model based on additive manufacturing was developed, and the investment casting process was optimized. These improved the production efficiency and the accuracy of the blade, to a certain extent.
3. The rationale, effectiveness and practicability of the method are verified by using the three-coordinate measuring machine to detect the blade’s surface.

Although the GTB processed by this method meet the requirements of surface quality and accuracy, in the future, it will be necessary to further strengthen the research on the performance test, mechanical strength, reliability and service life of GTB under different complex working conditions. The follow-up research work mainly includes the following aspects:

1. The casting material used in this paper is a heat-resistant cast steel material of the A297HK model. The performance of other casting materials (including superalloys) will be further studied to provide theoretical guidance and technical support for the design and manufacture of GTB that can adapt to more working conditions.
2. Under different complex working conditions, the performance test and structural analysis of the manufactured blades will be carried out to understand the possible problems and deficiencies in the performance parameters and reliability of the blades in practical applications. The detection of a single crystal or crystal-like structure will be carried out to further improve the internal quality and dimensional accuracy of the casting.
3. The investment casting process of additive manufacturing ABS material for blade models will be further optimized, including fully baking the blade model to reduce the presence of slag inside the casting. At the same time, the influence of the shrinkage rate of the metal materials on the size of the blade castings during the pouring process will be studied to improve the surface quality and dimensional accuracy of the blade castings.

## Figures and Tables

**Table 1:** Relevant parameter settings for additive manufacturing.

Print Mode	Printing Material	Material Diameter d/(mm)	Packing Density	Storey Height ΔZ (mm)	Total Number of Layers
FDM	ABS	1.75	100%	0.2	742

**Table 2:** Molded line profile deviations of the blade cross section.

Parameter	Maximum Deviation (mm)	Minimum Deviation (mm)	Standard Deviation (mm)
Section B-B	0.086	−0.081	0.043
Section C-C	0.090	−0.088	0.057

## Data Availability

Sorry, we are unwilling to share the relevant data of this article. Because these data may contain sensitive information, such as patent information, trade secrets, etc.
